# Wideband performance analysis of ground-plane cloak designed with polarization-independent randomly patterned metamaterial

**DOI:** 10.1038/s41598-018-26849-9

**Published:** 2018-05-31

**Authors:** Hyunsoo Lee, Il-Suek Koh, Yongjune Kim, Yongshik Lee

**Affiliations:** 1 Inha University, Department of Electronic Engineering, Incheon, 22212 South Korea; 2 Center for Advanced Meta-Materials, Daejeon, 34103 South Korea; 3 Yonsei University, Department of Electrical and Electronic Engineering, Seoul, 03722 South Korea

## Abstract

A ground-plane cloak is designed based on the quasi-conformal mapping method to hide a perfectly conducting object. It is fabricated with a metamaterial, a mixture of a dielectric and air. Using the dielectric mixing formula, the required volume fraction is calculated for a designed refractive index of the cloak. To guarantee the statistical isotropy of the cloak structure, many small pixels are randomly connected to form the metamaterial. A three-dimensional printing machine is used to implement the whole designed cloak structure. The performance of the cloak is experimentally analyzed over a wide frequency range for both independent polarizations. The measurement is also validated by numerical full-wave simulations. Because the quasi-conformal mapping generates unrealistic refractive indices, less than unity, those are removed. The effect of the truncation is experimentally observed and theoretically analyzed by the ray-tracing method.

## Introduction

Because a metamaterial can be easily synthesized to provide any electromagnetic properties that do not exist in nature^1^, metamaterials have been studied in the last decade and applied to many electromagnetic applications such as antennas, absorbers, lenses, and cloaking devices^2–5^. Special attention has been paid to a ground-plane cloak that can hide an object inside the cloaking medium. The cloak medium is inhomogeneous, and its relative permittivity or refractive index profile can be generated by the transformation optics (TO) technique^6–8^. The TO technique is based on the coordinate transformation, which changes the electromagnetic wave propagation path inside the medium by varying the refractive index. The computed refractive index profile is not easily implemented mainly due to two reasons. First, the values of the index fluctuate significantly, so that some values become very large for its implementation. The other is that the refractive index becomes anisotropic. To overcome such problems, a new coordinate transformation was introduced, known as quasi-conformal mapping (QCM)^9–12^. The QCM maintains the local orthogonality during the coordinate transformation process. Hence, the anisotropy of the cloaking medium can be minimized, and the values of the relative permittivity can vary in a smaller numeric range than for TO.

To implement the designed refractive index profile, metamaterial has usually been used because it can be easily fabricated with arbitrary relative permittivity or permeability. Many metamaterials are design based on a resonance phenomenon. This kind of metamaterial has the advantage of implementing very high relative permittivity. However, its operating frequency range becomes very narrow. In addition, the relative permittivity of the meta-cell is dependent on the polarization of the incident wave. Some meta-cell structures such as drilled hole and fishnet have been introduced to relieve the polarization dependency^13–19^. Metasurface can be an alternative approach to implement a cloak^20–22^, where the phase-shifting resonant elements on the surface compensate the phase difference and restore the wavefront of the reflective field. Hence, the volume cloak can be replaced by the thin surface.

The QCM generates a relative permittivity profile depending on the shape of the object hidden by the ground-plane cloak while the permittivity is fixed as unity. In the literature, a drilled hole or I-shaped material has been adopted for the ground-plane cloak structure^13–15^. The dielectric with an arbitrary refractive index can be easily synthesized by mixing dielectrics with different relative permittivities. The resulting permittivity value can be theoretically estimated by some known mixing formulas^23–26^.

Recently, a meta-cell has been proposed, which mixes a dielectric and air^27^. Thus, the effective relative permittivity of the mixture ranges from 1 to the value of the relative permittivity of the dielectric. Using a random pattern, moreover, the synthesized dielectric can be statistically isotropic. Owing to the complicated shape of the pattern, a three-dimensional (3D) printing machine is used to fabricate the meta-cell. The characteristic of the meta-cell is experimentally analyzed^27^.

In this paper, we construct a ground-plane cloak consisting of the randomly patterned meta-cell. The design method and fabrication of the ground-plane cloak are addressed. Experimental verification and results are also provided. The effective operating frequency bandwidth of the implemented cloak is theoretically analyzed and experimentally verified.

## Results

### Design and fabrication

**Figure 1:**
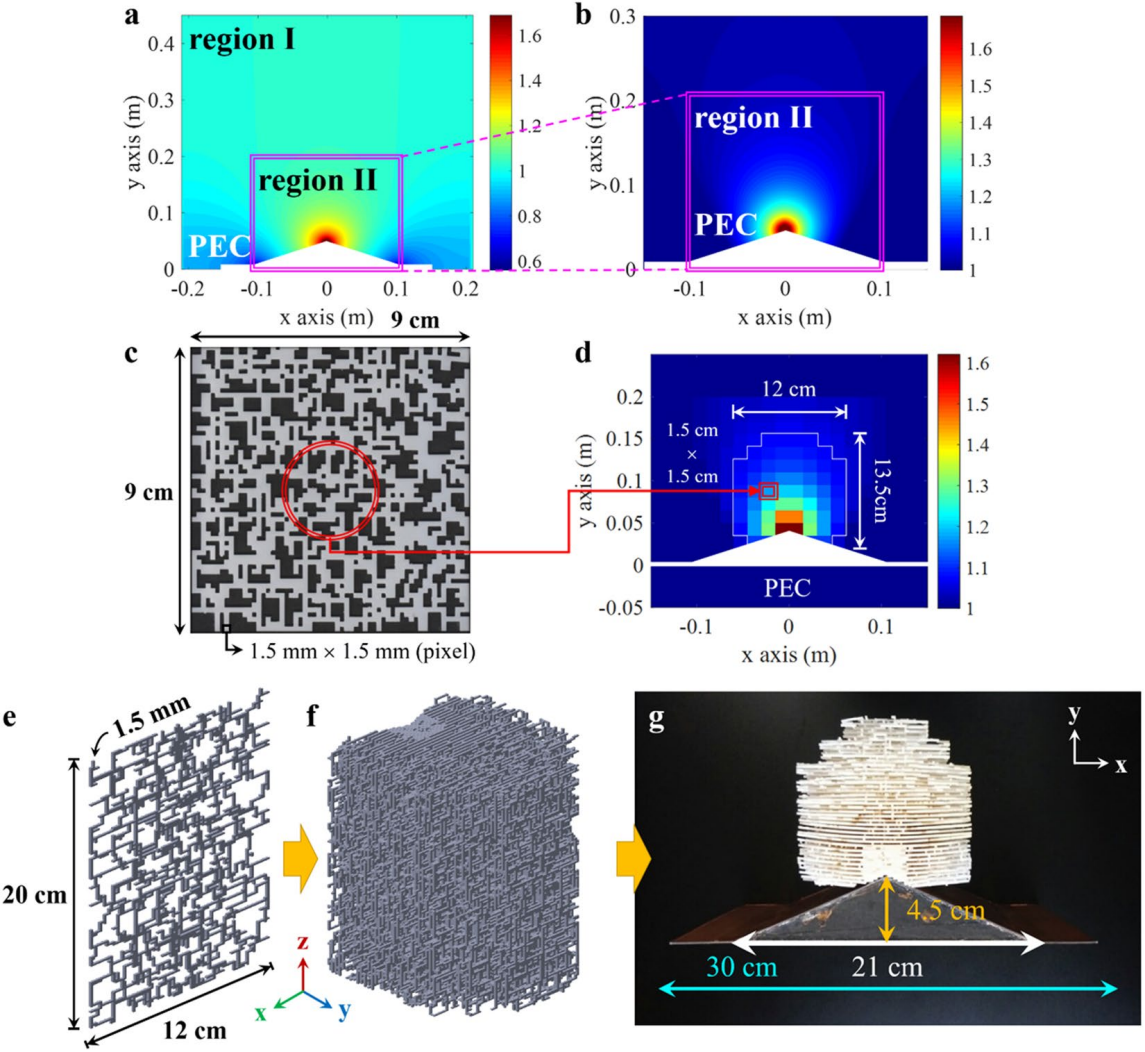
(**a**) Continuous refractive index profile of 2D ground-plane cloak, wherein the designed profile includes refractive index less than unity. The refractive index greater than unity is truncated in (**b**). (**c**) One sample layer of the meta-cell whose volume fraction is uniformly 0.314 and the corresponding refractive index is 1.174. (**d**) Discretized refractive index profile of the 2D ground-plane cloak. Each unit cell will be realized by using the meta-cell in (**c**). (**e**) CAD model of one planar layer for 3D ground-plane cloak. (**f**) CAD model of the full 3D ground-plane cloak. It consists of 90 layers of random patterns in (**e**). (**g**) Fabricated 3D ground-plane cloak by using 3D printer.

Figure 1 shows the process of the construction for 3D ground-plane cloak. It starts from considering two-dimensional (2D) ground-plane cloak hiding an object on the ground plane in Fig. 1(a), where the cloak medium is an inhomogeneous dielectric. The ground plane and the object, a triangular bump, are both perfect electric conductors (PEC). The dimensions of the triangular bump are 21 cm (*x*) × 4.5 cm (*y*). The length of the ground plane is assumed to be 30 cm (*x*). The QCM is used to generate the refractive index ranging from 0.6 to approximately 1.7^9–12^. It is not easy to manufacture the material with a refractive index less than unity and simultaneously the unit permeability (region I in Fig. 1(a)), which is forced to be removed. Hence, region II in Fig. 1(b) can be implemented.

The inhomogeneous dielectric medium can be fabricated with a mixture of dielectrics with different refractive indices. In this paper, we mix a dielectric with a refractive index (*n*) 1.69 and air (*n* = 1). To manufacture a dielectric with a specific relative permittivity, the volume fraction of two dielectrics is important, which can be estimated by several mixing formulas such as Maxwell–Garnett and coherent potential^23–26^. This kind of new metamaterial can be synthesized by randomly connecting many small dielectric pixels as seen in Fig. 1(c). The pixel is a small cubic box, whose size is 1.5 mm × 1.5 mm × 1.5 mm. The density of the pixels can be determined by the required volume fraction for a specific relative permittivity. Figure 1(c) shows a fabricated sample of the randomly patterned metamaterial with a uniform volume fraction of 0.314 corresponding to *n* = 1.174. The white and black portions are the dielectric and air, respectively.

This kind of randomly patterned metamaterial has two major advantages. First, owing to the random shape of the metamaterial, the metamaterial becomes rotationally symmetric in a statistical sense. Therefore, the metamaterial can interact with both independent polarizations of the incident wave in identical fashion^27^. The second is that the synthesized relative permittivity can be constant over a wider frequency range than the conventional meta-cell based on the resonance^27^.

The designed cloak profile in Fig. 1(b) is discretized by 15 mm × 15 mm cells as seen in Fig. 1(d). The refractive index is estimated at the center point of each cell, and the required volume fraction is calculated based on the mixing formulas. The randomly patterned meta-cell can be used to realize each cell, but the resulting shape may be very complex. A 3D printer can fabricate this kind of complicated perforated structure. After the construction, many supporters should be eliminated, which are required during the fabrication procedure of a 3D perforated structure. Hence, we implement a cell with 10 planar layers. The dimensions of each layer are 12 cm (*x*) × 1.5 mm (*y*) × 20 cm (*z*) as shown in Fig. 1(e). Thus, along the *x*-direction, the relative permittivity is changed by varying the density of the pixels. Along the *y*-direction, the 10 layers are stacked to build one discretized cell. The CAD model of the one layer and realized whole cloak profile are shown in Fig. 1(e) and (f), respectively. A total of 90 layers are fabricated by a 3D printer, Project 6000 at Hankook Archive Inc., to form the complete cloak medium as seen in Fig. 1(g). The total dimensions of the implemented cloak structure are 12 cm (*x*) × 13.5 cm (*y*) × 20 cm (*z*).

The resolution and net build volume of the Project 6000 are 125 *μ*m (*x*) × 100 *μ*m (*y*) × 125 *μ*m (*z*) and 25 cm × 25 cm × 25 cm, respectively. The fabrication procedure for the cloak profile is as follow. First, the CAD file of the designed profile is converted into a stereo lithography file format and imported to the 3D printing machine. The acrylic polymer is then deposited and cured by the ultraviolet lamp constructing the designed profile layer by layer. The refractive index and loss tangent of the polymer are 1.69 and 0.03, respectively. Because the loss tangent is very small, we can ignore the loss in practical applications^28–32^. Hence, the refractive index of the metamaterial can ideally vary from 1 to 1.69.

**Figure 2:**
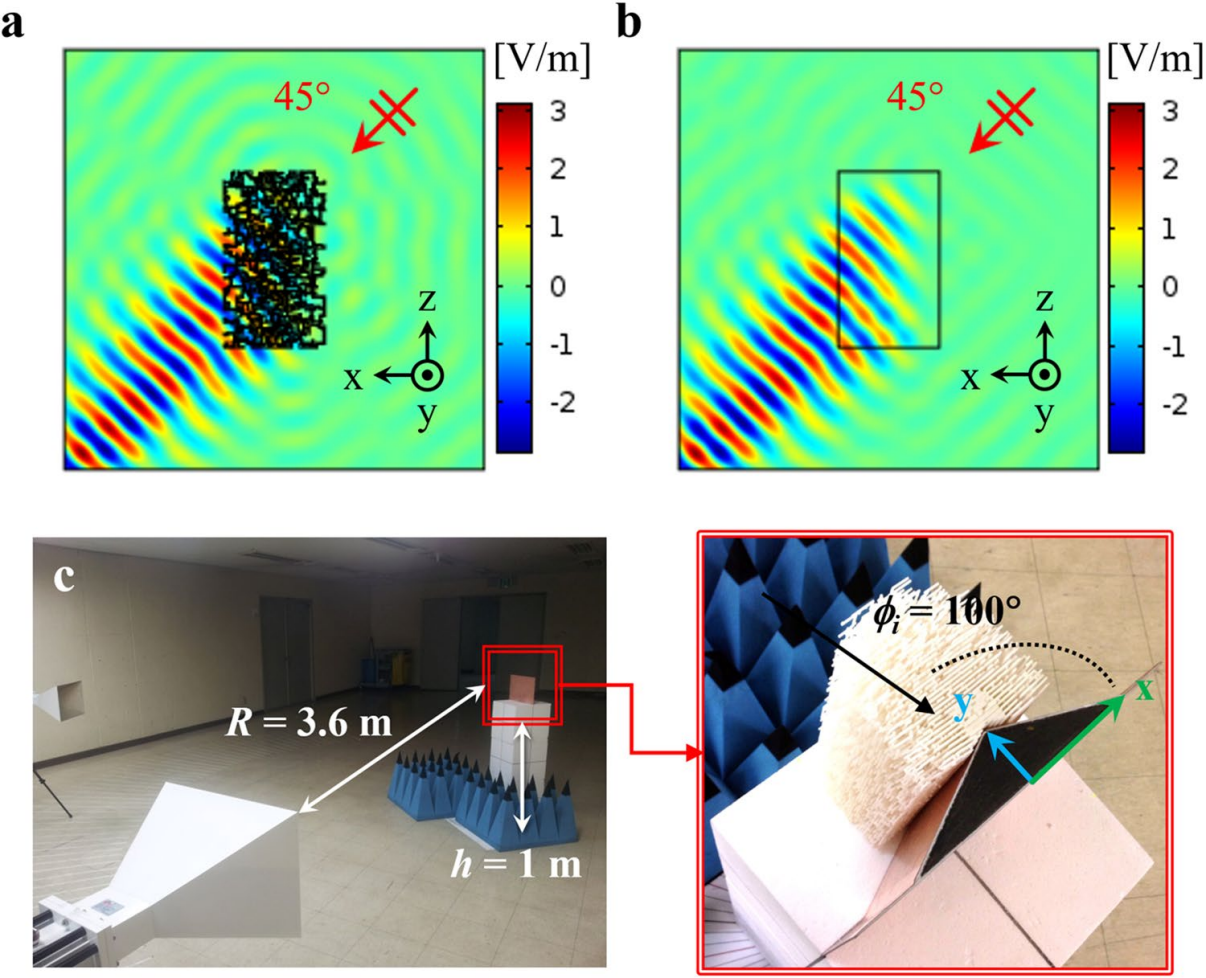
(**a**) 2D scattering simulation for *v*-polarized plane wave with incident angle 45° by rectangular cylinder whose cross section is one of the 90 layers in Fig. 1(f). (**b**) The same simulation with continuously varying inhomogeneous cross section. (**c**) Measurement setup of bistatic scattering for cloak experiment. The cloak is supported by a Styrofoam podium. Incident angle is set to 100°, and both polarizations are used.

The whole randomly patterned metamaterial structure (3D) cannot be numerically simulated owing to its complexity and size. Hence, we consider a 2D scattering simulation to show that the randomly patterned structure can mimic the scattering of the original inhomogeneous medium. Two simulations are compared. One is a scattering by a rectangular cylinder whose cross section is the random pattern of one layer as seen in Fig. 2(a), and the other one is a scattering by an inhomogeneous rectangular cylinder whose refractive index (original designed value) continuously varies along the cross section as seen in Fig. 2(b). The layer shown in Fig. 2(a) is one of the 90 layers in Fig. 1(f). The finite element method (FEM) in COMSOL Multiphysics is used to compute the scattering. A 10 GHz *v*-polarized plane wave is incident on the cylinders, and the incident angles is 45°. The dimensions of the rectangular cylinder are 12 cm (*x*) × 20 cm (*y*). Two simulation results, scattered field, are in good agreement as observed in Fig. 2(a) and (b). Therefore, the randomly patterned metamaterial can provide scattering almost identical to that by the inhomogeneous medium. For the same reason, the inhomogeneous dielectric object (Fig. 1(d)) can be used for a 3D numerical verification in HFSS instead of the real fabricated metamaterial structure (Fig. 1(g)).

### Experimental results

To experimentally verify the performance of the designed ground-plane cloak, we perform a bistatic scattering measurement over wide frequencies from 5 to 15 GHz. Figure 2(c) shows the measurement setup. Two standard horn antennas are used as a transmitter and a receiver. Plane wave is transmitted at 100°, and the receiver is swept from 20° to 140° with a 2° sampling angle. The scattered wave is most distorted at 100° by the triangular bump, so that the incident angle is chosen. At 10 GHz, the distance between the antennas and the cloak is *R* = 3.6 m or 120 *λ*_0_, where *λ*_0_ is the free-space wavelength at 10 GHz. The heights of the antennas and cloak structure are both *h* = 1 m or 33.3 *λ*_0_. The cloak is supported by a Styrofoam cylinder. The time-gating technique is used to remove the reflected field from the background such as a wall or a floor. For the calibration, a PEC sphere with radius of 15 cm and a 30 cm (*x*) × 20 cm (*y*) rectangular PEC plate are used.

**Figure 3:**
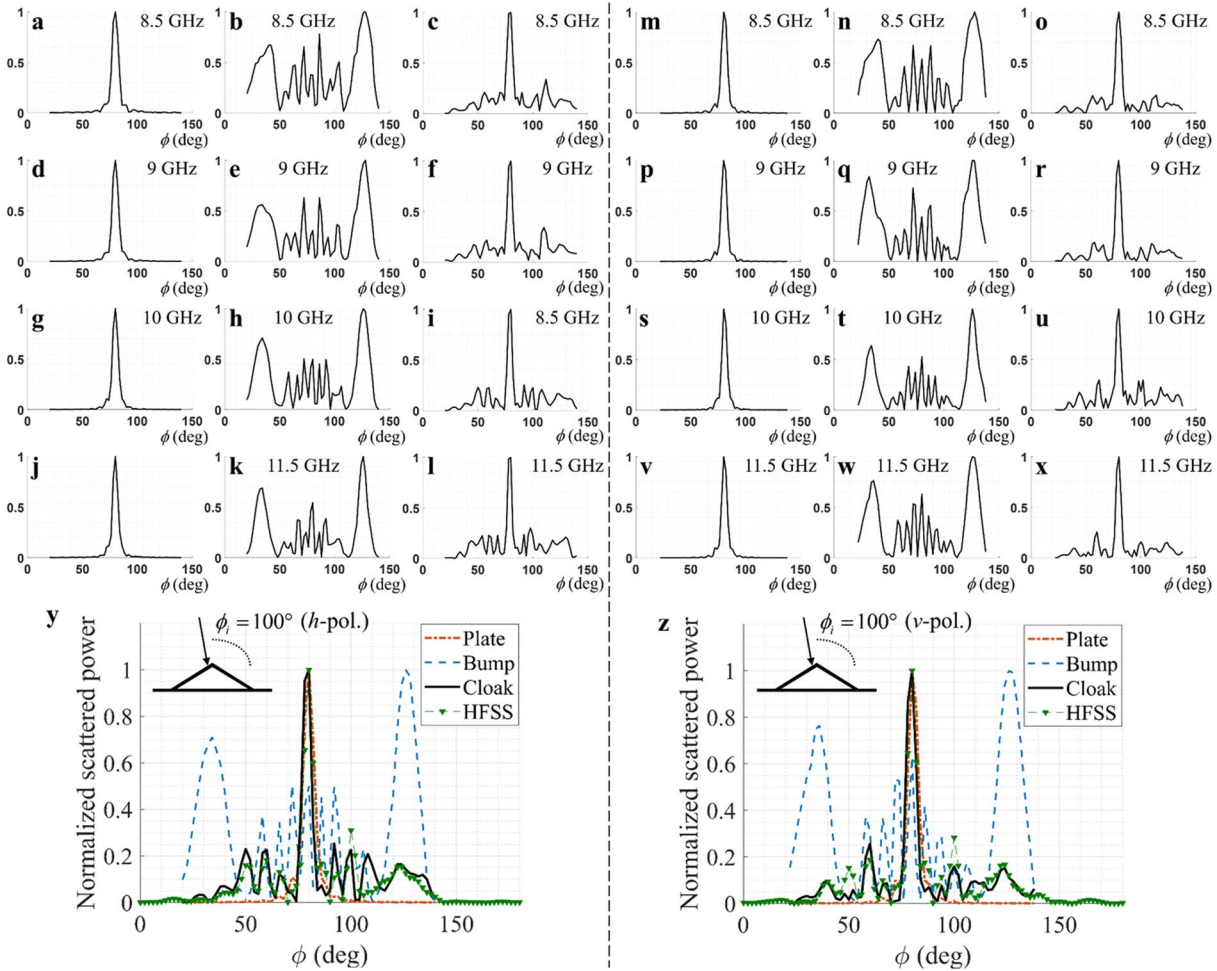
Normalized powers of scatterings as a function of observation angle. The incident angle is set to 100°. Scatterings for *h*-polarized incident wave at 8.5 GHz (**a**–**c**), 9 GHz (**d**–**f**), 10 GHz (**g**–**i**), and 11.5 GHz (**j**–**l**) frequencies are compared. Each column corresponds to scattering for *h*-polarization by ground plane (**a**,**d**,**g**,**j**), triangular bump on the plane (**b**,**e**,**h**,**k**), and cloak covering the bump (**c**,**f**,**i**,**l**), from the left to right. Scatterings for *v*-polarized incident wave at 8.5 GHz (**m**–**o**), 9 GHz (**p**–**r**), 10 GHz (**s**–**u**), and 11.5 GHz (**v**–**x**) frequencies are compared. Each column corresponds to scattering for *v*-polarization by ground plane (**m**,**p**,**s**,**v**), triangular bump on the plane (**n**,**q**,**t**,**w**), and cloak covering the bump (**o**,**r**,**u**,**x**), from the left to right. Especially, the normalized powers of scatterings at 10 GHz are compared with HFSS simulation for (*y*) *h*-polarization and (z) *v*-polarization.

Figure 3 shows the powers of scatterings as a function of observation angle for four different frequencies (8.5 GHz, 9 GHz, 10 GHz, and 11.5 GHz) and both independent polarizations (horizontal (*h*) and vertical (*v*) polarizations). The power is normalized by its peak value. For a simple comparison, Fig. 3(a–h) and j–l are the plots of the scattered powers for *v*-polarized incident wave at 8.5 GHz, 9 GHz, 10 GHz, and 11.5 GHz, respectively. Figure 3(a,d,g,f) show the scattered powers when only the ground plane exist. Figure 3(b,e,h,k) are the scattered powers by the triangular bump on the ground plane. The scattered powers by the cloak covering the bump are shown in Fig. 3(c,f,i,l). In the similar way, the scattered powers for *v*-polarized incident wave are measured and depicted in Fig. 3(m–x). It can be observed in Fig. 3(a–l) and (m–x) that the scattering without the cloak structure is totally different from the scattering by the ground plane. However, with the cloak structure, the scattering by the ground plane can be effectively restored regardless of the polarizations, especially along the specular direction. Figure 3(y,z) shows the comparisons of the normalized powers of scatterings by the ground plane, the bump on the ground plane, and the cloak covering the bump for *v*- and *h*-polarizations at 10 GHz. To verify the measurement results for the cloak, a full-wave simulation using HFSS at 10 GHz is added in Fig. 3(y,z). The measured data excellently agree with the HFSS simulation results. These comparisons clearly verify that the scattering by the bump can be matched with that of the ground plane by using the cloak structure.

**Figure 4:**
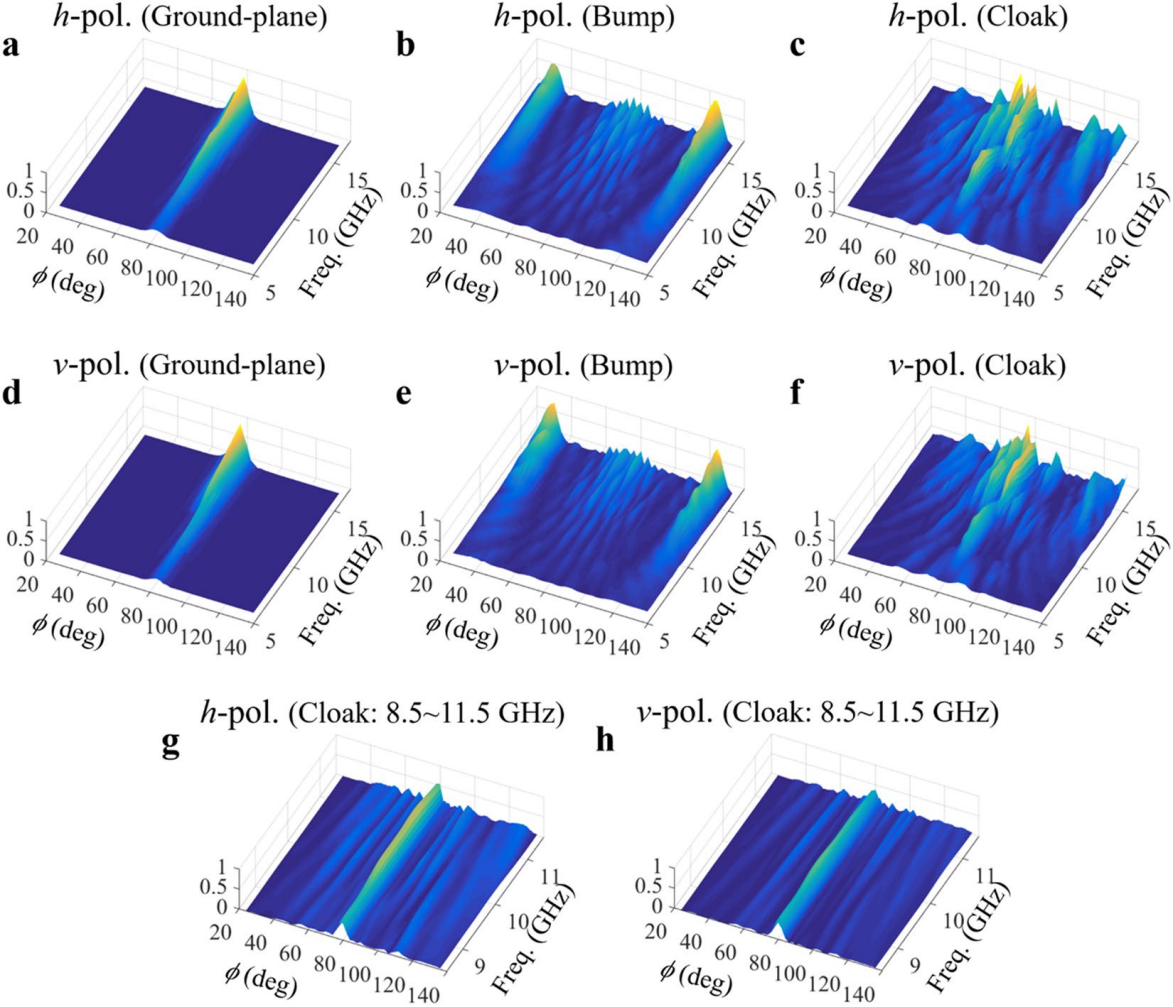
Measured scatterings for wider frequency range from 5 to 15 GHz. Scattering with -polarization by (**a**) ground plane, (**b**) triangular bump on the plane, and (**c**) cloak covering the bump are compared. Scattering with *v*-polarization by (**d**) ground plane, (**e**) triangular bump on the plane, and (**f**) cloak covering the bump are also compared. Especially, the measured scatterings by cloak for truncated frequency range from 8.5 to 11.5 GHz with (**g**) *h*-polarization and (**h**) *v*-polarization are illustrated.

Figure 3 shows only four frequencies results for brief comparisons. For a wider frequency range, the measured and normalized scattered powers are plotted in Fig. 4. The frequency ranges from 5 to 15 GHz. Figure 4(a–c) show the results for the *h*-polarized incident wave. Figure 4(d–f) are the plots for the *v*-polarization. The scatterings by the ground plane are shown in Fig. 4(a) and (d). Figure 4(b) and (e) are the scattered powers by the triangular bump on the ground plane. The results for the cloak structure are shown in Fig. 4(c) and (f). Figure 4(g) and (h) show the results of Fig. 4(e) and (f) in a frequency range from 8.5 to 11.5 GHz. From this results, it is confirmed that the cloaking structure restores the scattering by the bare bump. However, the operating frequency bandwidth is limited from 8.5 to 11.5 GHz. The working fractional bandwidth can be calculated such as $|{f}_{high}-{f}_{low}|/{f}_{center}$ × 100, which is approximately 30% fractional bandwidth.

**Figure 5:**
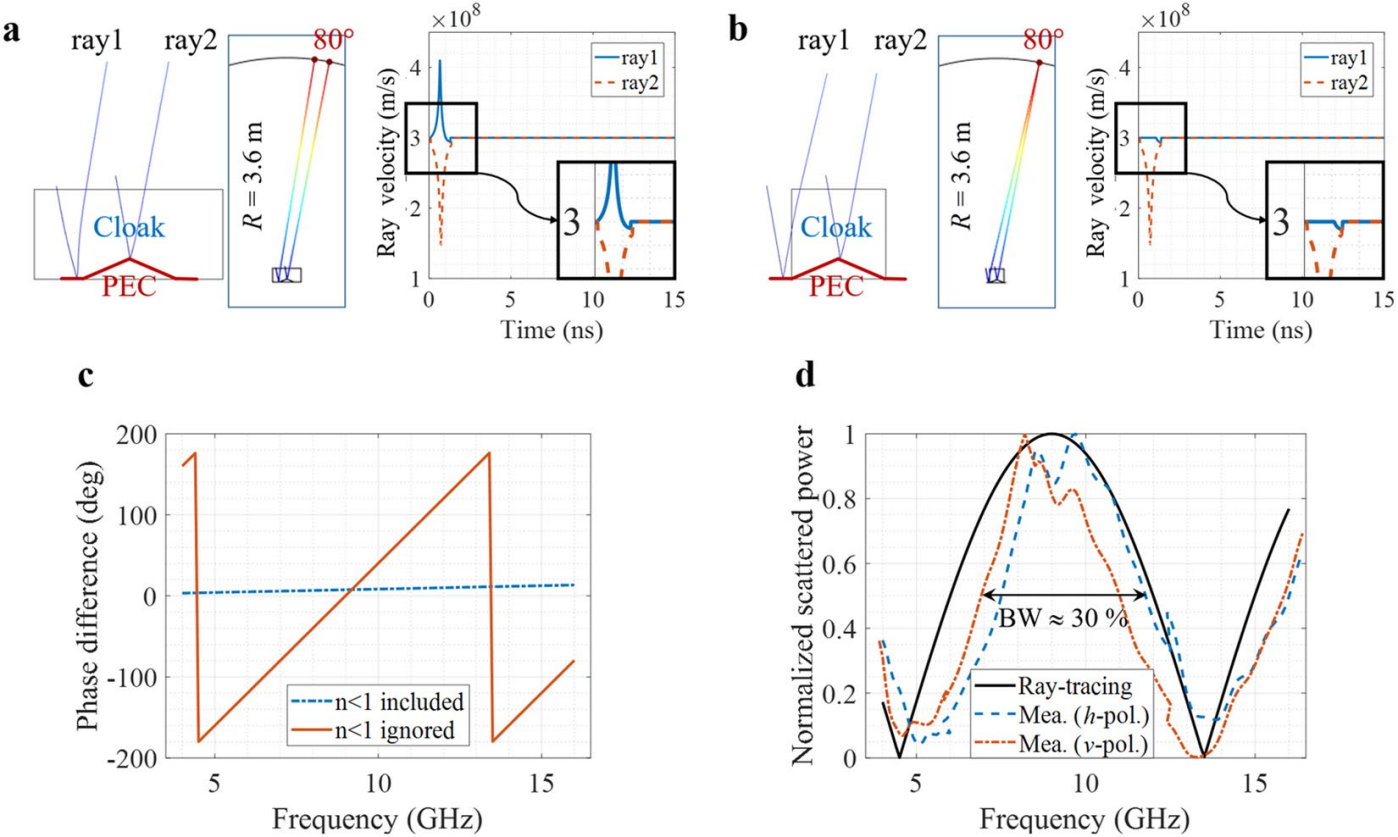
Comparison of velocities of two rays propagating inside cloak medium with (**a**) originally designed refractive index profile and (**b**) truncated profile. The refractive index outside the cloak depicted in (**a**,**b**) is almost 1. (**c**) Phase differences between ray 1 and ray 2 for the complete (original) and truncated profiles. (**d**) Estimated power variation by two-ray model in specular direction compared with measurement results for both polarizations

**Figure 6:**
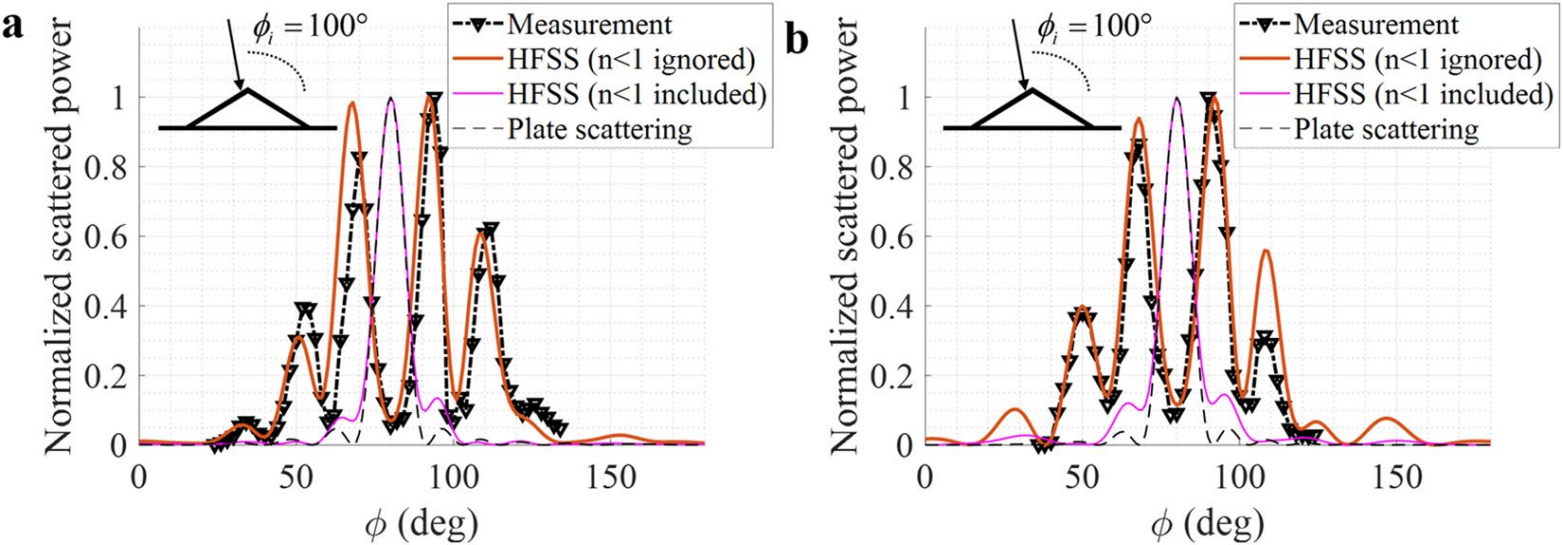
Comparison of normalized received powers between measurement and simulation result at 5 GHz for (**a**) *h*-polarization and (**b**) *v*-polarization.

**Figure 7:**
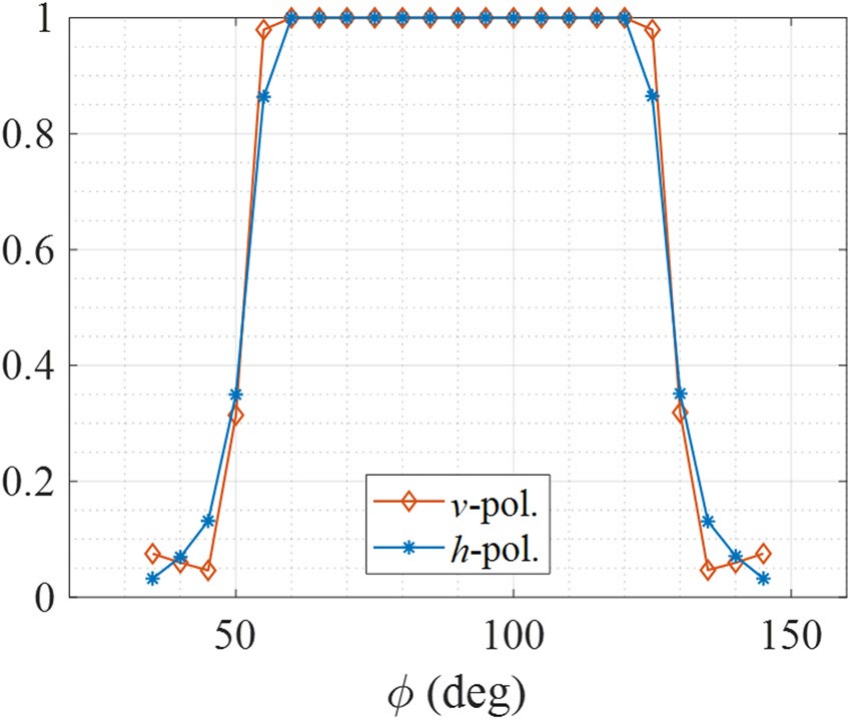
Ratios of normalized scattered power with and without cloak structure for two polarizations at specular direction at 10 GHz.

To physically explain the reason for the reduction of the cloaking bandwidth, the ray-tracing method is used. Figure 5(a) (or Fig. 1(a)) is the originally designed region including *n* < 1, and Fig. 5(b) (or Fig. 1(b)) is the implementable region (*n* > 1). We consider two rays for a simple explanation: ray 1 propagates mostly in region I; ray 2 propagates mainly in region II as seen in Fig. 5(a,b). The propagation path length of ray 1 should be different from the originally designed path length due to the removal of the *n *< 1 region. The simulated velocities of both rays are also shown in Fig. 5(a,b), which are inversely proportional to the refractive index. This path difference can cause ray 1 to arrive at a different observation point as seen in Fig. 5(b), which can disperse the power along the observation angles as seen in Fig. 4(e) and (f). Figure 5(c) shows the phase differences in the specular direction (=80°) between the two rays for the complete cloak and truncated cloak structures. Ideally, the phase difference should be constant over the whole frequency range, but owing to the truncation, the phase difference varies as a function of frequency. Based on Fig. 5(c), it can be estimated that the truncated cloak can work at approximately 10 GHz, where the phase difference is similar to that for the complete cloak. Figure 5(d) shows the comparison between the measured powers of scatterings for both polarizations and the estimated power variation by using the two-ray model in specular direction. The simple ray model can accurately estimate the effective operating bandwidth. The ray trajectory is exported from COMSOL Multiphysics, and the phase of each ray can be calculated as:

1 $$\frac{2\pi f}{{c}_{0}}\int n(s)ds$$

where *c*_0_ is the free-space speed of light. To show that the cloak does not operate properly outside the operating frequency bandwidth, the measurement results are compared with the HFSS simulation at 5 GHz for both polarizations in Fig. 6. The frequency of 5 GHz is selected to show the worst case because the rays are out-of-phase at 5 GHz as shown in Fig. 5(c). The two results are in excellent agreement. When the *n* < 1 region is not removed, the simulation results show the ideal cloak response, scattering by the plate only as seen Fig. 6(a,b). Therefore, the 3D printer-fabricated cloak structure consisting of the randomly patterned metamaterial operates over a wide but limited frequency range and independently of the incidence polarizations. Furthermore, over the whole incidence angle, the ratio of the normalized scattered power with and without the cloak is calculated at the specular direction at 10 GHz by using HFSS. As seen in Fig. 7, the *n* < 1 region still limits the incidence angle, but the cloak structure works for a wide incidence angles, $\varphi$ = 55°~125°.

## Discussion

A ground-plane cloak medium is designed based on QCM to hide a PEC triangular bump on a PEC ground plane. The cloak medium is an inhomogeneous dielectric whose refractive index value varies from 0.6 to approximately 1.7. Hence, the designed cloak profile can be fabricated with the randomly patterned metamaterial, a mixture of a dielectric and air. The volume fraction of the dielectrics can generate the required refractive index and can be computed by using the mixing formulas. Many small dielectric pixels are randomly connected to realize the designed randomly patterned metamaterial. It can operate independently of polarizations and has wider frequency bandwidth. Using a 3D printer, the designed cloak is fabricated. The *n* < 1 portion of the cloak structure is truncated.

A wideband experiment is performed from 5 to 15 GHz. The bistatic scattered fields are measured by the ground plane, the triangular bump on the ground plane, and the camouflaged triangular bump by the cloak for both independent polarizations. Several experiment results are numerically verified by the HFSS simulations. For the numerical simulations, the exact random pattern cannot be considered due to the complexity. Therefore, the scattering is computed for the original inhomogeneous refractive index profile. The experiment shows that the cloak can restore the scattering by the ground plane, especially along the specular direction independently of the incidence polarizations.

In addition, it is experimentally observed that the implemented cloak profile can operate in a limited bandwidth from 8.5 to 11.5 GHz (30% fractional bandwidth). Using the ray tracing method, it is theoretically shown that the *n* < 1 portion removal results in the phase shift of the ray propagation inside the truncated region. Hence, the destructive interference occurs outside the operating frequency range in specular direction. This ray based explanation is experimentally and numerically verified also.

## Methods

We implement the refractive index and the required volume fraction for the designed metamaterial based on the mixing formulas^23–26^. Among them equation (2) is simple and popular:

2 $${\varepsilon }_{eff}={\varepsilon }_{b}+\frac{\sum _{k=a,b,c}\frac{\varphi ({\varepsilon }_{i}-{\varepsilon }_{b})[{\varepsilon }_{b}+v({\varepsilon }_{eff}-{\varepsilon }_{b})]}{3[{\varepsilon }_{b}+v({\varepsilon }_{eff}-{\varepsilon }_{b})+{N}^{k}({\varepsilon }_{i}-{\varepsilon }_{b})]}}{1-\sum _{k=a,b,c}\frac{\varphi {N}^{k}({\varepsilon }_{i}-{\varepsilon }_{b})}{3[{\varepsilon }_{b}+v({\varepsilon }_{eff}-{\varepsilon }_{b})+{N}^{k}({\varepsilon }_{i}-{\varepsilon }_{b})]}}$$

where ${\varepsilon }_{b}$, ${\varepsilon }_{i}$, and ${\varepsilon }_{eff}$ represent the relative permittivity of the background, inclusion, and effective media, respectively. $\varphi$ is the volume fraction of the inclusion in the mixture, ${N}^{k}$ is the depolarization factor, and ${N}^{a,b,c}\mathrm{=1/3}$ for a spherical (or cubical) inclusion^33^. Equation (2) contains three different formulas based on the value of *v*: Maxwell-Garnett formula when *v* = 0, Polder van Santen formula when $v=1-{N}^{k}$, and the coherent potential formula when *v* = 1. By mixing a dielectric with air, we can obtain the relative permittivity or refractive index of the metamaterial ranging from 1 to 1.69 (the refractive index of the acrylic polymer used in the 3D printer). Randomness plays a key role in ensuring the statistical homogeneity of the permittivity and results in polarization independence. For a robust metamaterial structure, however, the dielectric particles or cubical pixels should be not only randomly located but also fully connected.

The design procedure of the random pattern is as follows: First, calculate the volume fraction for the required relative permittivity based on (2). Set the size of dielectric inclusion or cubical pixel (1.5 mm × 1.5 mm × 1.5 mm), which usually is slightly larger than the ideal resolution of the 3D printer (125 *μ*m (*x*) × 100 *μ*m (*y*) × 125 *μ*m (*z*)). Then choose a point randomly on the boundary of one meta-cell, and generate a path in a random walk-like fashion until the path arrives the opposite boundary of the meta-cell. The dimensions of the meta-cell are 15 mm × 15 mm × 15 mm, therefore, one meta-cell can contain one thousand of cubical pixels at most. The volume fraction can be estimated by counting the number of pixels on the generated path in the meta-cell. The extra path will be added if the number of pixels on the path don’t meet the required volume fraction. The extra path should be connected to the previously generated path. This can be done by checking whether there are any intersections between paths. If not, the extra path is discarded, and another path is attempted.

## References

[1] Cui, T. J., Smith, D. & Liu, R. *Metamaterials*: *Theory*, *Design*, *and Applications*. (Springer US, 2009).

[2] Zhu J, Eleftheriades GV (2009). A compact transmission-line metamaterial antenna with extended bandwidth. IEEE Antennas and Wireless Propagation Letters.

[3] Landy NI, Sajuyigbe S, Mock JJ, Smith DR, Padilla WJ (2008). Perfect metamaterial absorber. Physical Review Letters.

[4] Kundtz N, Smith DR (2010). Extreme-angle broadband metamaterial lens. Nature Materials.

[5] Schurig D, Mock JJ, Justice BJ, Cummer SA (2006). Metamaterial electromagnetic cloak at microwave frequencies. Science.

[6] Leonhardt. Optical conformal mapping. *Science* **312**, 1777–1780 (2006). 10.1126/science.1126493 16728596

[7] Pendry JB, Schurig D, Smith DR (2006). Controlling electromagnetic field. Science.

[8] Cai W, Chettiar UK, Kildishev AV, Shalaev VM (2007). Optical cloaking with metamaterials. Nature Photonics.

[9] Steinberg, S. *Fundamentals of Grid Generation*. (Taylor & Francis, 1993).

[10] Li J, Pendry JB (2008). Hiding under the carpet: a new strategy for cloaking. Physical Review Letters.

[11] Tang W, Argyropoulos C, Kallos E, Song W, Hao Y (2010). Discrete coordinate transformation for designing all-dielectric flat antennas. IEEE Transactions on Antennas and Propagation.

[12] Kallos E, Argyropoulos C, Hao Y (2009). Ground-plane quasicloaking for free space. Physical Review A.

[13] Liu R (2009). Broadband ground-plane cloak. Science.

[14] Ma H, Cui T (2010). Three-dimensional broadband ground-plane cloak made of metamaterials. Nature Communications.

[15] Ma H, Cui T (2010). Three-dimensional broadband and broad-angle transformation optics lens. Nature Communications.

[16] Jiang WX (2013). Broadband all-dielectric magnifying lens for far-field high-resolution imaging. Advanced Materials.

[17] Jiang WX (2016). Shaping 3D path of electromagnetic waves using gradient-refractive-index metamaterials. Advanced Science.

[18] Alici K, Ozbay E (2008). A planar metamaterial: polarization independent fishnet structure. Photonics and Nanostructures-Fundamentals and Applications.

[19] Menzel C, Paul T, Rockstuhl C, Pertsch T, Tretyakov S (2010). Validity of effective material parameters for optical fishnet metamaterials. Physical Review B.

[20] Ni X, Wong ZJ, Mrejen M, Wang Y, Zhang X (2015). An ultrathin invisibility skin cloak for visible light. Science.

[21] Yang Y (2016). Full-polarization 3d metasurface cloak with preserved amplitude and phase. Advanced Materials.

[22] Yang Y, Wang H, Yu F, Xu Z, Chen H (2016). A metasurface carpet cloak for electromagnetic, acoustic and water waves. Scientific reports.

[23] Sihvolva A, Kong J (1998). Effective permittivity of dielectric mixtures. IEEE Transactions on Geoscience and Remote Sensing.

[24] McLachlan D, Blaszkiewicz M, Newnham R (1990). Electrical resistivity of composites. Journal of the American Ceramic Society.

[25] Jylha L, Sihvolva A (2007). Equation for the effective permittivity of particle-filled composites for material design applications. Journal of Physics D: Applied Physics.

[26] Jones S, Friedman S (2000). Particle shape effects on the effective permittivity of anisotropic or isotropic media consisting of aligned or randomly oriented ellipsoidal particles. Water Resources Research.

[27] Lee H, Koh IS, Lee Y, Seo I (2016). Design of a wideband polarization- independent metamaterial with arbitrary relative permittivity based on the dielectric mixing theory. International Journal of Nanotechnology.

[28] Wu Z, Kinast J, Gehm M, Xin H (2008). Rapid and inexpensive fabrication of terahertz electromagnetic bandgap structure. Optics express.

[29] Karikkainen K, Sihvolva A, Nikoskinen K (2000). Effective permittivity of mixtures: numerical validation by the fdtd method. IEEE Transactions on Geoscience and Remote Sensing.

[30] Karikkainen K, Sihvolva A, Nikoskinen K (2001). Analysis of three-dimensional dielectric mixture with finite difference method. IEEE Transactions on Geoscience and Remote Sensing.

[31] Sihvolva A, Alanen E (1991). Studies of mixing formulae in the complex plane. IEEE Transactions on Geoscience and Remote Sensing.

[32] Sihvolva A (2002). How strict are theoretical bounds for dielectric properties of mixtures. IEEE Transactions on Geoscience and Remote Sensing.

[33] Yaghjian A (1980). Electric dyadic green’s functions in the source region. Proceedings of the IEEE.

